# Heat shock increases conjugation efficiency in *Clostridium difficile*^☆^

**DOI:** 10.1016/j.anaerobe.2016.06.009

**Published:** 2016-12

**Authors:** Joseph A. Kirk, Robert P. Fagan

**Affiliations:** Krebs Institute, Department of Molecular Biology and Biotechnology, University of Sheffield, Sheffield, S10 2TN, UK

**Keywords:** *Clostridium difficile*, PCR ribotype 027, Conjugation

## Abstract

*Clostridium difficile* infection has increased in incidence and severity over the past decade, and poses a unique threat to human health. However, genetic manipulation of *C*. *difficile* remains in its infancy and the bacterium remains relatively poorly characterised. Low-efficiency conjugation is currently the only available method for transfer of plasmid DNA into *C*. *difficile*. This is practically limiting and has slowed progress in understanding this important pathogen. Conjugation efficiency varies widely between strains, with important clinically relevant strains such as R20291 being particularly refractory to plasmid transfer. Here we present an optimised conjugation method in which the recipient *C*. *difficile* is heat treated prior to conjugation. This significantly improves conjugation efficiency in all *C*. *difficile* strains tested including R20291. Conjugation efficiency was also affected by the choice of media on which conjugations were performed, with standard BHI media giving most transconjugant recovery. Using our optimised method greatly increased the ease with which the chromosome of R20291 could be precisely manipulated by homologous recombination. Our method improves on current conjugation protocols and will help speed genetic manipulation of strains otherwise difficult to work with.

## Highlights

• Conjugation efficiency in *Clostridium difficile* can be increased through heat treatment of recipient *C*. *difficile*.
• Conjugation efficiency using an optimised heat treatment conjugation protocol is affected by media choice.
• Conjugation efficiency is improved when a heat treatment step is included, using a plasmid with a pBP1 replicon.
• Heat treatment improves conjugation efficiency in all *C*. *difficile* strains tested.

## 1 Introduction

*Clostridium difficile* poses a significant threat to human health and is the most common cause of antibiotic associated diarrhoea [1]. Both incidence and mortality has increased over the last decade. This is due, at least in part, to the emergence of epidemic lineages such as ribotype 027 and 078 [1]. Despite the increase incidence and severity of *C*. *difficile* infections, relatively little is known about the molecular basis of disease. However, an increased recognition of the importance of *C*. *difficile* in human health has led to an increased interest in this bacterium. Despite this, genetic manipulation of *C*. *difficile* remains in its infancy. One limitation in *C*. *difficile* research is the reliance on low-efficiency conjugation, as this is the only useable method for DNA transfer into *C*. *difficile* [2]. This is less of a problem when using strains with relatively high conjugation efficiencies such as the laboratory strain CD630. However, clinically relevant epidemic strains, such as the ribotype 027 strain R20291, display very low conjugation efficiencies. This makes genetic manipulation of these strains particularly difficult.

Conjugation efficiencies of other bacteria, such as *Campylobacter jejuni* and *Corynebacterium glutamicum*, have been shown to increase in response to heat treatment [3], [4]. Here we demonstrate that conjugation efficiency in a panel of *C*. *difficile* strains is increased in response to heat treatment and we provide an optimised conjugation protocol that will increase the ease with which the species may be genetically manipulated.

## 2 Materials and methods

### 2.1 Growth and handling of organisms

*C*. *difficile* strains, outlined in Table 1, were propagated either in TY broth without thioglycolate [5] or on BHI agar. Cultures were incubated at 37 °C in an anaerobic environment composed of 80% nitrogen, 10% hydrogen and 10% carbon dioxide. *Escherichia coli* was routinely grown in LB broth or on LB agar. *E*. *coli* strain CA434 (HB101 carrying R702) was used as the conjugation donor throughout. NEB5α (New England Biolabs) was used for cloning and plasmid propagation. Growth media was supplemented with chloramphenicol (15 μg/ml), thiamphenicol (15 μg/ml) or cycloserine (250 μg/ml) as appropriate.

### 2.2 Viability of *C*. *difficile* after heat treatment

*C*. *difficile* strain R20291 was grown overnight in TY broth. 200 μl samples were incubated at 44, 46, 48, 50, and 52 °C for 5, 15, 30, 45, or 60 min. An unheated control was included. Samples were then serially diluted and 10 μl of each dilution was spotted onto well-dried BHI plates and incubated overnight. Enumerations were performed in triplicate on biological duplicates. Colonies of *C*. *difficile* were counted and viability calculated as CFU/ml.

### 2.3 Plasmid conjugations

A previously described and widely used conjugation protocol was used as the starting point for development of our improved method [2]. 200 μl samples of *C*. *difficile* overnight cultures were heated, as above, and incubated at 37 °C for 2 min. 1 ml of overnight *E*. *coli* conjugant donor (CA434) culture was harvested by centrifugation at 4000*g* for 2 min and transferred into the anaerobic workstation. *E*. *coli* cell pellets were then gently resuspended in 200 μl of heat treated or untreated *C*. *difficile* culture. This mixed cell suspension was then pipetted onto well-dried, non-selective agar plates (10 × 10 μl spots) and allowed to dry. BHI agar was used routinely but BHIS (BHI agar supplemented with 0.1% (w/v) cysteine and 0.5% (w/v) yeast extract), TY [5] and Brazier’s (Brazier’s CCEY media, 1% (v/v) defibrinated horse blood, 4% (v/v) egg yolk emulsion) agar were also tested. All solid media contained 1.5% agar. Conjugations were then incubated for 8–24 h following which growth was harvested using 900 μl of TY broth, serially diluted and spread on plates containing either cycloserine (for total *C*. *difficile* CFU counts), or cycloserine and thiamphenicol (to select for transconjugants). Approximate conjugation efficiency was then calculated as transconjugant CFU/total *C*. *difficile* CFU. These experiments were performed using biological duplicates with technical triplicates. Statistical significance of these results was determined using either individual student t-tests or in combination with one way analysis of variance (ANOVA), performed using GraphPad Prism 6. *P* values of<0.05 were considered statistically significant.

### 2.4 Creation of a chromosomal *divIVA-SNAP* fusion mutant and fluorescence microscopy

1.2 kb upstream and downstream of the 3′ region of *divIVA* were amplified using RF439 (CGTAGAAATACGGTGTTTTTTGTTACCCTACTGTAGCAATATTAAATTCTACAAATG) with RF440 (CAGCAGCTGCCTCGAGTTCTAAAGTTGTAGCAGCTTC) and RF443 (CCAGGACTTGGGTAAGCTTGTAATTTGTTTATTTTTTATG) with RF444 (GGGATTTTGGTCATGAGATTATCAAAAAGGACATTTGATGGTAAAGTCCATG) respectively. A *C*. *difficile* codon-optimised SNAP-tag coding sequence was cloned from pFT46 [6] using RF441 (GCTACAACTTTAGAACTCGAGGCAGCTGCTGAT) and RF442 (AAACAAATTACAAGCTTACCCAAGTCCTGGTTTCC). pMTL-SC7215 [7] was linearized using RF311 (TAGGGTAACAAAAAACACCG) and RF312 (CCTTTTTGATAATCTCATGACC). These fragments were then combined using a combination of SOEing PCR [8] and Gibson assembly [9] using the HiFi DNA assembly kit (New England Biolabs). The resulting plasmid (pJAK050) was then conjugated into *C*. *difficile* strain R20291 and mutants selected using the method previously described [7]. Correct insertion of the SNAP-tag coding sequence into the chromosome was confirmed by PCR using RF445 (GGTTTAAGAGGGTATAGAGATG) and RF545 (CGAGTTATAAATCGCGTTACCACC). For fluorescent labelling of DivIVA-SNAP, 1 ml of exponentially growing R20291 and R20291 *divIVA-SNAP* was incubated anaerobically with 250 nM SNAP TMR-star (New England Biolabs) for 30 min before slides were prepared for standard fluorescence microscopy, or lysates prepared. Proteins in the lysates were resolved on 12% SDS-PAGE gels and visualised using a BioRad ChemiDoc MP imaging system. Slides were visualised using a Nikon Ti Eclipse inverted epifluorescence microscope.

## 3 Results and discussion

### 3.1 Heat treatment of R20291 increases conjugation efficiency

**Fig. 1:**
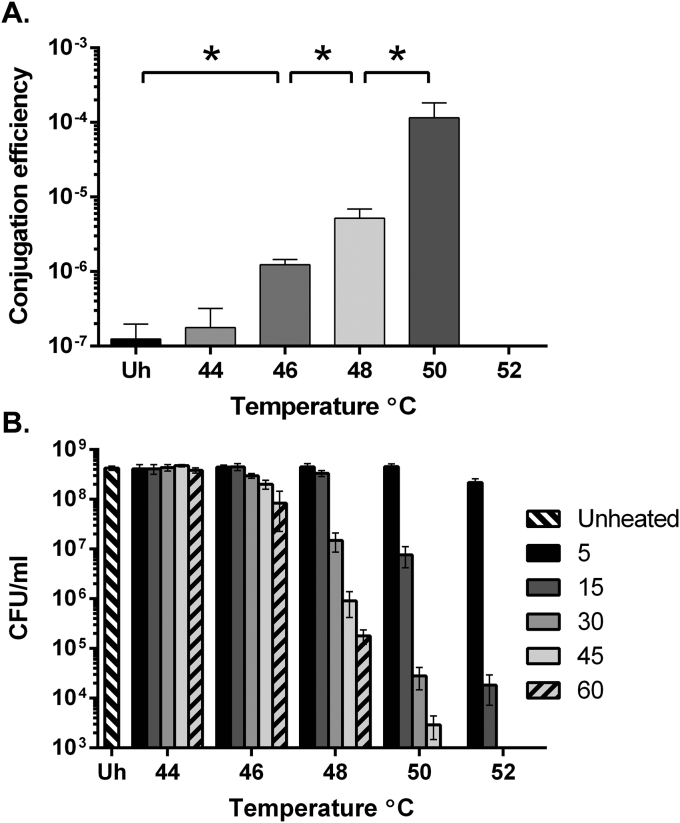
Effect of heat treatment on conjugation efficiency and viability of *C*. *difficile* strain R20291. **(A)** Conjugation efficiency of unheated and heat treated (44–52 °C for 15 min) *C*. *difficile* strain R20291. Asterisks (*) denote statistical significance between heat treatment conditions (P < 0.05). Each bar represents the mean and standard deviation of data collected from experiments performed in triplicate using biological duplicates. **(B)** Viability of R20291 after heat treatment at varying temperatures (44–52 °C) and incubation times (5–60 min). Each bar represents the mean and standard deviation of data collected from experiments performed in triplicate using biological duplicates.

*C*. *difficile* strain R20291 was chosen for these experiments due to its clinical relevance and relatively low conjugation efficiency. The effect of heat treatment on conjugation efficiency was tested. R20291 cultures were heated to 44–52 °C for 15 min prior to conjugation with the *E*. *coli* donor CA434 bearing the plasmid pRPF185 [10]. pRPF185 was chosen as it is a commonly used plasmid in *C*. *difficile* research and contains the native *C*. *difficile* replicon pCD6. A clear increase in conjugation efficiency was observed with increasing temperatures, from 46 °C to 50 °C (Fig. 1A). However, no transconjugants were observed when treated at 52 °C for 15 min. Additionally, although conjugation efficiency was increased at 50 °C relative to 48 °C, lower total CFUs and transconjugant CFUs were recorded. To test the effect of the heat treatment on *C*. *difficile* viability, cultures were heated to 44–52 °C for 5–60 min and viable cells enumerated (Fig. 1B). Viability was unaffected by incubation at 44 °C but there was a significant reduction in viability at temperatures above this and viability further decreased with increasing incubation time. Based on these data it is clear that the total number of transconjugants recovered is a compromise between increasing conjugation efficiency and decreasing viability. Although heat treatment at 52 °C for 15 min resulted in recovery of no transconjugants, this treatment did reduce viability by more than four orders of magnitude. As shorter heat treatments minimised the impact on viability, conjugation was tested following a 52 °C heat treatment for 5 min. This treatment almost doubled the recovery of transconjugants compared to the next best condition (approx. 2800 CFU/ml following 52 °C, 5 min compared with 1500 CFU/ml following 50 °C, 15min). These conditions were used as standard for the remainder of the experiments described below unless otherwise stated.

### 3.2 Conjugation efficiency is affected by media choice

**Fig. 2:**
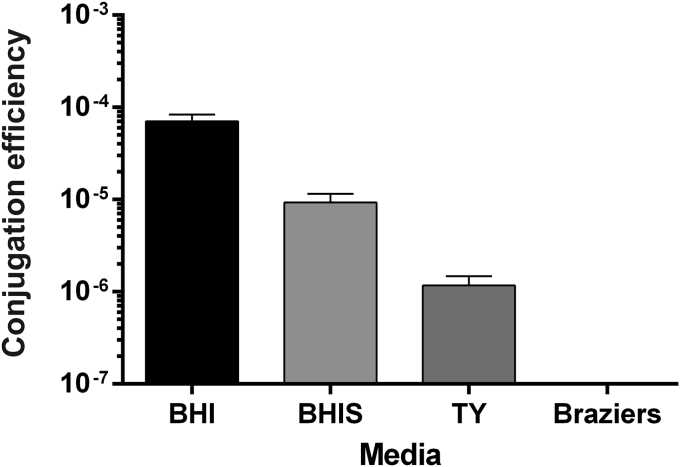
Effect of media choice on conjugation efficiency of *C*. *difficile* strain R20291 after heat treatment. BHI, BHIS, TY and Brazier’s agar were tested in 8 h conjugations following a 52 °C, 5 min heat treatment. Use of Brazier’s agar resulted in the recovery of no transconjugants. Each bar represents the mean and standard deviation of data collected from experiments performed in triplicate using biological duplicates.

There appears to be no standard media choice for *C*. *difficile* conjugations in the current literature. For this reason, the effect of media choice on conjugation efficiency, after heat treatment, was investigated. Conjugations were performed on BHI, BHIS, TY, and Braziers agar (Fig. 2). The highest conjugation efficiency was recorded when conjugations were performed using BHI agar. Although conjugation efficiency was lower when using BHIS relative to TY, higher numbers of transconjugants were recovered when using TY due to a higher total CFU count recorded when using this growth medium. Total *C*. *difficile* CFUs recovered from Brazier’s was the lowest of all tested media, and resulted in the recovery of no transconjugants. While this observation is unlikely to be due to the media having a direct effect on the actually conjugation process, it is likely that population equilibrium between *C*. *difficile* and *E*. *coli* is affected, which may in turn impact overall conjugation efficiency.

### 3.3 Heat treatment improves conjugation efficiency of a plasmid containing a non-native replicon

**Fig. 3:**
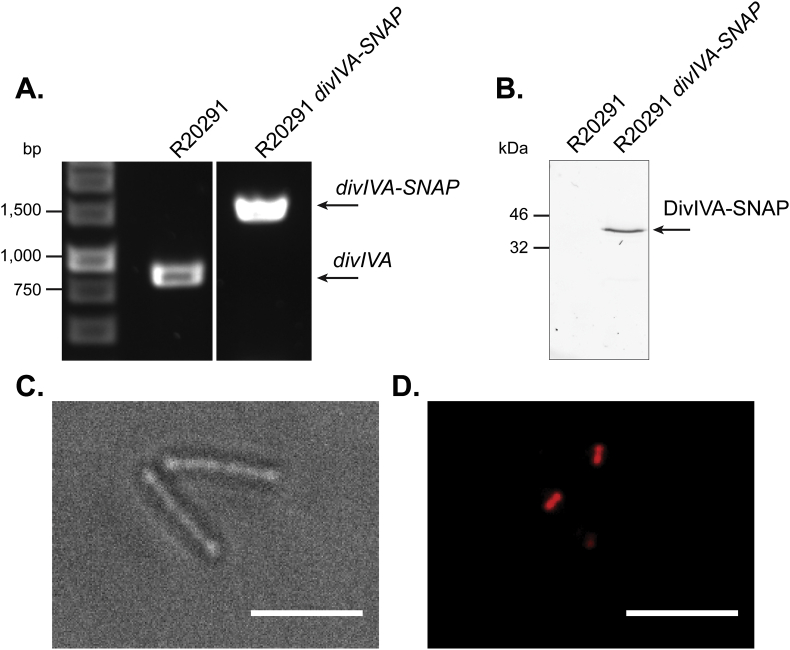
Precise manipulation of the R20291 genome accelerated by the use of optimised conjugation. **(A)** 0.8% Agarose gel showing PCR fragments amplified using primers flanking the SNAP-tag coding sequence insertion site in the chromosomal *divIVA* gene. The increase of approximately 550bp from 885 bp (R20291) to 1443 bp (R20291 *divIVA-SNAP)* suggests the correct insertion of the SNAP-tag coding sequence. **(B)** 12% SDS-PAGE gel imaged using a fluorescence imager, showing resolved lysates from SNAP TMR-star treated R20291 and R20291*divIVA-SNAP*. A band at approximately 40 kDa in the mutant corresponds to the addition of a SNAP-tag on DivIVA. **(C)** Brightfield and **(D)** fluorescence microscopy of exponentially growing R20291*divIVA-SNAP* stained with 250 nM SNAP TMR-star for 30 min, showing mostly septal and some polar localisation of fluorescence. This demonstrates successful modification of the R20291 genome. Scale bar represents 5 μm.

The precise manipulation of the genome of R20291 can be achieved through the use of the allele-exchange vector pMTL-SC7215. This pseudosuicide vector contains the pBP1 replicon, which replicates at a rate lower than that of the host cell chromosome. The result is non-exponential growth, limited by the rate of plasmid replication, when under antibiotic selection [7]. However, plasmids based upon this vector are difficult to conjugate into strain R20291 and the number of publications describing the use of this plasmid remains low. For this reason a pMTL-SC7215 based vector was created to add the SNAP-tag coding sequence to the chromosomal *divIVA* gene. This plasmid, pJAK050, was then used in heat treated and unheated conjugations. Due to the extremely low conjugation efficiency when using this plasmid, 24 h conjugations were performed following a 50 °C for 15 min heat treatment. No transconjugants were recovered using the standard unheated conjugation protocol. However, using our optimised heat treatment protocol large numbers of transconjugants were recovered, allowing a *divIVA-SNAP* mutant to be isolated in little over 2 weeks after the plasmid was constructed. Correct insertion of the SNAP-tag coding region into the *C*. *difficile* genome was confirmed by PCR (Fig. 3A). Fluorescence imaging of lysates of SNAP TMR-star treated R20291 *divIVA-SNAP* resolved by SDS-PAGE shows a band at approximately 40 kDa, the estimated size of DivIVA-SNAP (Fig. 3B). SNAP TMR-star treated R20291 *divIVA-SNAP* displayed mostly septal and some polar localisation of fluorescence (Fig. 3C and D), similar to DivIVA localisation observed in other bacterial species [11]. Taken together, these results suggest correct insertion of the SNAP-tag coding sequence into the *C*. *difficile* chromosome at the appropriate genetic locus.

### 3.4 Heat treatment increases conjugation efficiency in a panel of *C*. *difficile* strains

**Fig. 4:**
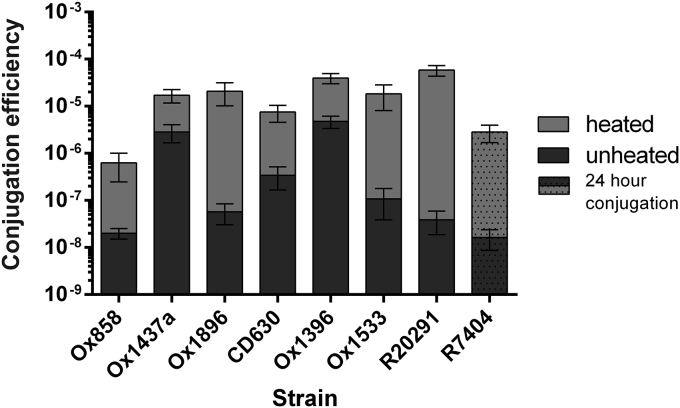
Effect of heat treatment on the conjugation efficiency of a panel of *C*. *difficile* strains. A panel of *C*. *difficile* strains was used in unheated (dark grey) and heat treated (light grey, 52 °C for 5 min) 8 h conjugations. Strain R7404 showed no significant increase in conjugation efficiency with this treatment and was therefore used in a 24 h conjugation after a more stressful heat treatment (50 °C for 15 min). Each bar represents the mean and standard deviation of data collected from experiments performed in triplicate using biological duplicates. All strains displayed a statistically significant (P < 0.05) increase in conjugation efficiency after heat treatment.

In order to determine whether the improved conjugation protocol was more widely applicable, conjugations were performed with pRPF185 on a panel of *C*. *difficile* strains. The strains tested represented a further 6 ribotypes and the majority of *C*. *difficile* clades and S-layer types [12]. All strains tested displayed an increase in conjugation efficiency after heat treatment, although the observed improvement was greatest for strain R20291 (Fig. 4). Although efficiency was improved, heat treatment did not result in a significant increase in the number of transconjugants recovered for strains Ox1396 and Ox1437a. This is likely due to a decrease in viability following heat treatment and may be improved by extending the conjugation time. Using the standard 52 °C for 5 min heat treatment, conjugation efficiency into strain R7404 was too low to accurately measure after an 8 h conjugation and no significant increase in conjugation efficiency was observed when conjugations were extended to 24 h. R7404 conjugation was then tested after incubation at 50 °C for 15 min followed by a 24 h incubation. This resulted in a significant increase in both conjugation efficiency (Fig. 4) and in the number of transconjugants recovered. These results indicate that our heat treatment conjugation protocol is broadly applicable to the majority of *C*. *difficile* strains and may be further optimised for specific applications.

## 4 Conclusions

*C*. *difficile* shows considerable strain-to-strain variation in conjugation efficiency. Conjugation efficiency is improved in a panel of strains, representing the majority of clades and S-layer types, through heat treatment of the recipient *C*. *difficile* prior to conjugation. Conjugation efficiency is dependent on the media choice on which the conjugation is performed, with the highest number of transconjugants being recovered from BHI agar in this study. Using an optimised heat treatment conjugation protocol significantly improved the ease in which a pMTL-SC7215 based plasmid was introduced into R20291, facilitating chromosomal manipulation of the R20291 genome by homologous recombination. Heating the recipient *C*. *difficile* to 52 °C for 5 min prior to conjugation proved to be optimum for an 8 h conjugation, although conjugation efficiency can be increased by coupling more stressful heat treatments with longer conjugation times.

## Figures and Tables

**Table 1:** *C*. *difficile* strains used in this study including their respective clade, ribotype, and S-layer type.

*C*. *difficile* strain	Clade	Ribotype	S-layer type
Ox858	1	029	2
Ox1437a	1	026	5
Ox1896	1	018	6
630	1	012	7
Ox1396	1	012	8
Ox1533	1	129	10
R20291	2	027	4
R7404	4	017	7b
